# Ultrasonication-Assisted Green Synthesis and Physicochemical and Cytotoxic Activity Characterization of Protein-Based Nanoparticles from *Moringa oleifera* Seeds

**DOI:** 10.3390/nano14151254

**Published:** 2024-07-26

**Authors:** Amany Abd El-Shafy Abd El-Kader Nafeh, Ibrahim Mohamed Abd El-Aleem Mohamed, Mohamed Frahat Foda

**Affiliations:** 1 Department of Biochemistry, Faculty of Agriculture, Benha University, Moshtohor, Toukh 13736, Egypt; 2 National Key Laboratory of Crop Genetic Improvement, College of Life Science and Technology, Huazhong Agricultural University, Wuhan 430070, China

**Keywords:** *Moringa oleifera*, protein-based nanoparticles, ultrasonication, desolvation, THP-1 cells, cytotoxicity

## Abstract

*Moringa oleifera* (*M. oleifera*) is globally recognized for its medicinal properties and offers high-quality, protein-rich seeds. This study aimed to explore the potential of *M. oleifera* seeds as a significant source of protein-based nanoparticles (PBNPs) using the ultrasonication technique after desolvation and to evaluate their cytotoxicity in the human leukemia cell line (THP-1) for the first time. The properties of the PBNPs were confirmed by dynamic light scattering (DLS), transmission electron microscopy (TEM), scanning electron microscopy (SEM), energy-dispersive X-ray spectroscopy (EDX), X-ray diffraction (XRD), and Fourier-transform infrared spectroscopy (FT-IR). The extracted protein from moringa seed cake flour had a significant protein content of 54.20%, and the resulting PBNPs had an average size of 134.3 ± 0.47 nm with a robust zeta potential of −43.15 mV. Notably, our study revealed that PBNPs exhibited cytotoxic potential at high concentrations, especially against the THP-1 human leukemia cell line, which is widely used to study immunomodulatory properties. The inhibitory effect of PBNPs was quantitatively evidenced by a cytotoxicity assay, which showed that a concentration of 206.5 μg mL^−1^ (log conc. 2.315) was required to inhibit 50% of biological activity. In conclusion, our findings highlight the potential of *M. oleifera* seeds as a valuable resource in the innovative field of eco-friendly PBNPs by combining traditional medicinal applications with contemporary advancements in protein nanotechnology. However, further studies are required to ensure their biocompatibility.

## 1. Introduction

*Moringa oleifera* (*M. oleifera*) is globally recognized as a highly advantageous plant, often called the “miracle tree” owing to its exceptional nutritional and therapeutic properties. *M. oleifera* is the most widely cultivated species within the Moringa genus of the Moringaceae family, containing an extensive array of bioactive components across various plant parts [1]. These components include proteins, flavonoids, saponins, phenolic acids, tannins, isothiocyanates, lipids, minerals, and vitamins, which contribute to their multifaceted and valuable health-promoting attribute [2,3]. Additionally, *M. oleifera* seeds exhibit various pharmacological potential and health benefits, including antimicrobial, antioxidant, antidiabetic, anticancer, hepatoprotective, cardioprotective, antihypertensive, anti-inflammatory, and immunostimulatory properties [4,5]. *M. oleifera* also contains biologically active protein with molecular weights ranging from 3.4 to 20 kilodaltons (kDa), depending on the amino acid composition and structure [6,7].

Moringa protein extract is a robust alternative to animal proteins that contain all essential amino acids [8]. Research conducted on the amino acid composition of Moringa seed protein revealed a favorable hydrophobic amino acid ratio, balanced distribution of polar and non-polar amino acids, and proportional equilibrium between negatively and positively charged amino acids [9,10]. The significant protein content of the seed cake, combined with its appropriate amino acid composition, low molecular weight, and remarkable functional properties, make it a promising candidate for numerous applications, particularly as a base for producing nanoparticles.

Furthermore, the seed cake remaining after the oil extraction operation comprises a significant proportion of high-quality protein (approximately 42–52%) [11,12], an important biocomponent that must be utilized. Generally, seed proteins have a higher proportion of albumin and globulin compared to prolamin and glutelin. β-sheet is the predominant secondary conformation present in isolated proteins. Therefore, studying protein function is essential for the development of novel protein components [13]. Green nanotechnology has significantly revolutionized various fields, particularly the production of protein-based nanoparticles (PBNPs), enhancing their performance and broadening PBNP applications [14]. These developments offer opportunities to create materials capable of addressing significant systematic challenges [15,16]. Recently, protein-based nanostructures have transformed nanomedicine by becoming popular for delivering hydrophilic and hydrophobic drugs as well as bioactive compounds [17,18]. Their subcellular size allows them to penetrate tissues through capillaries and to be absorbed by cells [19].

Proteins used in nanoparticles can be classified into two main categories: animal and plant proteins. Compared to animal proteins, plant proteins are more affordable, readily available, and easier to purify. Moreover, the hydrophobic nature of plant proteins is advantageous because it allows crosslinking of toxic chemicals [20,21]. Proteins are distinguished from other plant polymers by their many biological roles, including antioxidant activity, antifungal activity, antibacterial activity, and immune system support [22]. Moreover, plant proteins can modify surfaces and activate various biomolecules, thus opening the horizon for numerous applications [23]. Sources of plant proteins for PBNPs include corn (zene), wheat (gliadin), sunflowers, pea protein, Moringa seeds, and soy proteins. PBNPs can also be obtained from protein-containing plants [24].

Protein nanoparticles are relatively simple to prepare and can be produced through a robust, cost-effective, and eco-friendly synthesis process using fewer chemicals compared to the production of other types of nanoparticle synthesis [25]. The desolvation method is widely used to produce protein nanoparticles in medicine, biotechnology, and food science [26]. The desolvation or coacervation technique is preferred for producing PBNPs, owing to its simplicity and ability to yield smaller particles. This technique reduces protein solubility in aqueous solutions by adding a desolvating agent such as ethanol or acetone, leading to phase separation [27,28]. The desolvating agent alters protein conformation and reduces its solubility, resulting in protein nanoprecipitate formation. Subsequently, the residual protein, crosslinker, and solvents were eliminated, and protein-based nanoparticles were dispersed in a selected solvent [28,29].

Ultrasonication is highly effective for various purposes when preparing PBNP solutions. These objectives include dispersing protein nanoparticles in base fluids to prevent agglomeration, reducing the PBNP size within the fluid, activating particle surfaces, and increasing the electrostatic stability of the molecules in the solution, which is represented by the zeta potential. Zeta potential is a measure of the electrostatic stability of the particles in a solution. Particles with high zeta potentials (negative or positive) are electrically stable in solution, whereas particles with low zeta potentials tend to coagulate or flocculate, which can lead to poor physical stability [30,31,32].

Studies have shown that PBNPs exhibit numerous properties in addition to their larger size and increased surface area. These attributes transform conventional materials, offering exciting possibilities for various applications [33]. PBNPs possess desirable characteristics such as biological origin, biodegradability, non-toxicity, immunocompatibility, wide availability, ease of preparation, and the ability to undergo surface modifications through reactive groups such as thiols, amines, and carboxyl groups. These surface modifications enable coupling bond formation, highly efficient drug loading, and targeted delivery to specific tissues and organs, thereby reducing systemic toxicity and increasing cellular uptake [20,34]. These favorable attributes make PBNPs highly appealing for numerous applications in nanobiotechnology and nanomedicine [15,35]. Cytotoxicity assays are critical initial biological evaluations and screening procedures for PBNPs to elucidate their potential for medical applications [36]. The THP-1 cell line, widely utilized in immune- and inflammation-related research, aligns well with the prospective biomedical uses of PBNPs [37].

In this study, we synthesized PBNPs from Moringa seed cake for the first time using an ultrasonication-assisted technique following a coacervation approach. The properties of the resulting PBNPs were investigated using dynamic light scattering (DLS), transmission electron microscopy (TEM), scanning electron microscopy (SEM), energy-dispersive X-ray analysis (EDX), X-ray diffraction (XRD), and Fourier-transform infrared spectroscopy (FT-IR). Additionally, we assessed the in vitro inhibitory effect of these PBNPs on the human leukemia cell line (THP-1) as a unique model to investigate and estimate the immunomodulatory effects of biological compounds.

## 2. Materials and Methods

### 2.1. Materials

Seeds of high-quality *M. oleifera* were obtained from the National Research Center (NRC) of El-Tahrir Street, Dokki, Cairo, Egypt. Spectra/Por1 dialysis membrane with 6–8 kDa and 0.5 kDa molecular weight cut-off, (MWCO), sodium dodecyl sulfate (SDS), tris-HCl buffer, β-mercaptoethanol, and Brilliant Blue were from Sigma Chemicals (St. Louis, MO, USA). The human leukemia monocytic cell line (THP-1) horseradish peroxidase (HRP) and ELISA kits were purchased from ATCC^®^, Manassas, VA, USA. Hydrochloric acid, for chemical usage, was supplied by Merck Millipore Chemicals (Temecula, CA, USA). The remaining chemicals used, namely hexane (95%), sodium hydroxide, and ethanol (99.9%), were acquired from reputed companies in Egypt. All the chemicals and solvents used were of the highest purity available.

### 2.2. Instrumentation

Thermolyne Benchtop Muffle Furnace (Model FB1315M), Soxhlet, Accuplate Hotplate Stirrer (5 × 7 Ceramic Top. from Labnet International, Inc., Corning, NY, USA), pH meter (Adwa AD1030), centrifuge (with High-Speed Rotor—Z36 HK), mortar, pestle, mesh sieve (0.15 mm), lyophilizer (Telstar LyoQuest), probe sonicator (model VCX 750, USA), DLS (Nicomp 380 ZLS), high-resolution TEM (HR-TEM) (JEOL, JEM-2100, Tokyo, Japan), SEM (Minifix 600 regaco), HR-SEM (Hitachi S-4700), FT-IR Bruker VERTEX 80 (Germany), balance (Radwag AS 220/C/2), and drying oven (RUMO) were made in Egypt. Microplate ELISA reader (FLUOstar OPTIMA, BMG LABTECH GmbH, Ortenberg, Germany) was obtained elsewhere.

### 2.3. Preparation of the M. oleifera Seed

Dry pods with ripe seeds were harvested in autumn, starting in September 2021/2022, from *M. oleifera* trees at the National Research Center in Egypt. The seeds were extracted from the pods and characterized by their brown peel color, wings, and spherical shape with a slight angle opposite to the hilum, presenting a small, linear, and not prominently raised shape. After removing the husk, the seeds were ground using a mortar and pestle and then sieved through a 0.15 mm mesh to eliminate coarse particles and achieve flour uniformity. The resulting particle sizes ranged from 0.1 to 0.15 mm.

### 2.4. Preparation of Defatted Moringa Seed Flour (DMSF)

To remove fats, oils, and waxes from moringa seed flour, we used a Soxhlet extractor with hexane (95%). Subsequently, the defatted flour was air-dried at room temperature for approximately 24 h and then finely ground. DMSF was stored in an airtight container until further use.

### 2.5. Proximate Composition

The assessment of moisture percentage, ash, total protein, fat, and crude fiber content followed the Association of Analytical Communities (AOAC) protocols (1995) [38]. Finally, the carbohydrate content was calculated from the difference.

The moisture, ash, and lipid content were estimated at the Biochemistry Department, Faculty of Agriculture, Benha University. Meanwhile, protein and fiber content were estimated at The National Research Center (NRC). Details of the proximate composition procedure are provided in the Supporting Information (SI) Section.

### 2.6. Preparation of Moringa Protein Isolate (MPI)

The following isolation steps were performed at the Biochemistry Department, Faculty of Agriculture, Benha University. Moringa protein was isolated from the seeds using an alkaline–acid extraction and isoelectric precipitation method based on the report by González Garza and Nancy Gisela et al., with some modifications [38]. Briefly, protein extraction from DMSF was performed at pH 11 in a 1:20 ratio (fine: alkaline solution). The alkaline solution was NaOH (1 N), and the pH was adjusted using HCl (2 N), followed by magnetic stirring for 2 h at room temperature, filtration through white gauze, and centrifugation at 4 °C (10,000 rpm for 15 min) to remove the cake residue [39]. The supernatant was acidified to pH 4 with (2 N) HCl and stored in a refrigerator at 4 °C for 24 h for protein precipitation. The acidified mixture was then centrifuged (10,000 rpm for 20 min) at 4 °C. These pH levels were chosen after several preliminary experiments to verify the optimal pH for extraction and the corresponding pH for protein precipitation. The precipitate was washed in a dialysis tube (6–8 kDa, MWCO) in an ice bath for 2 h using a magnetic stirrer. The temperature was monitored every 15 min to maintain the temperature below 10 °C by adding more ice, changing the dialysis solution (distilled water), and dialysis for another 2 h. On the other hand, protein isolates were rapidly frozen at −18 °C, then lyophilized and kept at −20 °C until further analysis. Protein content was determined using the Kjeldahl method (%N × 6.25) at the NRC [40].

### 2.7. Preparation of Protein-Based Nanoparticles (PBNPs)

PBNPs were prepared using a dissolution technique called nanoprecipitation, with ultrasonication adjustments. Briefly, the PBNP solution was prepared by resuspending the MPI with deionized water in the ratio of 5 g:0.1 L, 5 g:0.175 L, and 5 g:0.25 L (MPI/deionized water). The pH of the solution was adjusted to 9 by (1 N) NaOH. The protein solution was then equilibrated for 3 h at room temperature and stored overnight at 4 °C for 8 h to hydrate the protein molecules. Ethanol was then used as a self-assembling agent for protein molecules at a rate of 4 mL ethanol/1 mL protein solution. Ethanol was added dropwise to the MPI solution at a ratio of 1 mL min^−1^ using a German burette instead of the custom apparatus used by Jahanban-Esfahlan [41]. The MPI solution was purified by centrifugation at 10,000 rpm for 20 min at 4 °C. The precipitate was then collected and dissolved in an appropriate amount of distilled water.

Following the introduction of ethanol, adding two different concentrations of MPI/deionized water (5 g:0.1 L and 5 g:0.175 L), resulted in noticeable aggregate formation and precipitation during preparation, suggesting a significant particle size increase. This observation reflects the results reported by Hong, S. et al. (2020), who noted that higher protein concentrations led to larger nanoparticles [25]. Consequently, these two concentrations were excluded from further experiments. The remaining tests were conducted at a concentration of 5 g:0.25 L.

The purification process was performed using a dialysis tube (0.5 kD, MWCO) for 4 h with water to be changed every 2 h (as a washing solution). Dialysis helped purify the resulting nanoparticles and reduced aggregation [42]. All previous steps were conducted at the Biochemistry Department, Faculty of Agriculture, Benha University. Subsequently, ultrasonication of the sample was performed using a sonication probe (model VCX 750, USA) with a frequency of 20 KHz, amplitude of 20%, power output of 450 W, and 20 min duration at NRC to loosen the aggregates and obtain smaller nanoparticles [43]. The nanosuspensions obtained were freeze-dried to produce a fine powder of PBNPs.

**Scheme 1:**
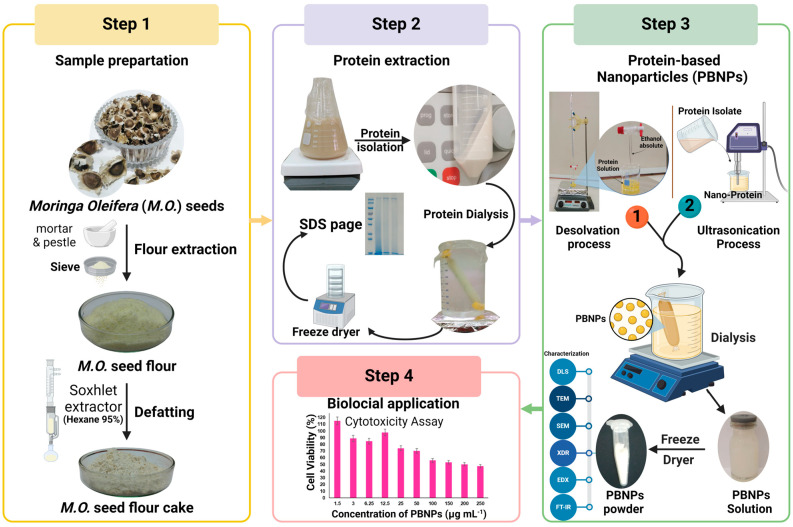
Schematic diagram of the synthesis and application of *M. oleifera* seed protein-based nanoparticles.

Additionally, a comprehensive schematic diagram shows a step-by-step procedure for producing PBNPs from *M. oleifera* seeds, which were then tested in cytotoxicity assays. The first procedure involved processing the seed samples, which included grinding to powder, extracting the flour, and then eliminating the fat to yield a residual seed cake. Subsequently, alkaline acid extraction and isoelectric precipitation were used. Dialysis procedures were then performed, followed by lyophilization for protein isolation. PBNPs were engineered in the third phase in two steps: desolvation and ultrasonication. The nanoparticles were then dialyzed and freeze-dried to obtain a flexible powder or stable colloidal solution. SDS-PAGE was employed to determine the molecular weights of the PBNPs prior to and following nanodilution. The biocompatibility of these PBNPs was thoroughly investigated using cytotoxicity and cytokine assays, which are critical steps in determining their applicability for medical and bioengineering applications, as presented in Scheme 1.

### 2.8. Molecular Weight of MPI and PBNPs

The molecular weights of MPI (crude protein) and PBNPs before and after ultrasonication were determined using sodium dodecyl sulfate and polyacrylamide gel electrophoresis (SDS-PAGE). Sample suspensions (4 mg mL^−1^ protein content) were boiled in SDS sample buffer (50 mM Tris-HCl, pH 6.8, 2% SDS, 0.1% Brilliant Blue, and 10% glycerol) containing 100 mM β-mercaptoethanol for 5 min. An aliquot of 10 μL was loaded onto a polyacrylamide gel consisting of a 15% solvent gel and a 5% stacking gel. The samples were separated by electrophoresis on a 15% gel. Electrophoresis was initially conducted at 100 V for 10 min to concentrate the sample, followed by running at 160 V for 60 min to separate the proteins based on their molecular weights [44]. After running, the gel was stained using the Colloidal Coomassie Brilliant Blue G-250 Staining protocol [45,46]. The stained gel was scanned with a Chemil-magerTM 4400 (Alpha Innotech, San Leandro, CA, USA).

### 2.9. Characterization of MPI and PBNPs

#### 2.9.1. The Morphology and Particle Size

The morphology of the NPs was examined after sonication using TEM at the NRC. To observe the microstructure, high-resolution TEM (HR-TEM) was performed using a JEOL JEM-2100 instrument in Tokyo, Japan. Before imaging, the samples were appropriately diluted with deionized water, deposited on a carbon film-coated copper grating, and allowed to dry naturally at room temperature. Images were captured at various magnifications at an accelerating voltage of 200 kV. Furthermore, SEM was employed at the Faculty of Science, Benha University, to investigate particle morphology. Briefly, a thin layer of freeze-dried sample was applied to a carbon film. Images at various magnifications were captured using an accelerating voltage of 26 kV (Minifix 600 Regaco). Additionally, EDX was utilized to assess the elemental composition of the crude protein and nanoprotein.

The nanoparticle size and zeta (ζ) potential were determined before and after the ultrasonication process using dynamic light scattering (DLS) at the NRC. A DLS device (Nicomp 380 ZLS) was applied for size distribution and zeta potential analysis of the MPI and (PBNPs). The samples were analyzed at a constant angle of 90° and temperature of 25 °C. A dilution of 0.02 M phosphate buffer pH 7.0 was utilized to assess the zeta potential, considering that the zeta potential depends on the pH of the solvent and ion strength.

#### 2.9.2. X-ray Diffraction (XRD) Analysis

MPI and PBNP powders were pressed into stainless-steel sample holders. The structural properties of MPI and PBNPs were measured using an X-ray diffractometer (D8 Advance, Bruker, Karlsruhe, Germany) [47,48]. The 3KWX generator, operating at 40 kV and 40 mA from a regulated power source, emitted Cu-Kα radiation filtered through nickel with a wavelength of 1.5418 Å. A 0.04 mm anti-scatter slit was employed, accompanied by divergence, with receiving slits set at 1°. The sample holders from Bruker had a diameter of 25 mm and goniometer radius of 280 mm. Scanning across a 2θ range of 5° to 70° was performed at a speed of 1°/min.

#### 2.9.3. Fourier-Transform Infrared Spectroscopy (FT-IR)

The lyophilized protein isolates and lyophilized nanoproteins (after sonication) were evaluated by FT-IR spectroscopy Bruker VERTEX 80 (Ettlingen, Germany) at the Faculty of Science, Benha University. FT-IR spectra were acquired, and data were processed using the Origin software version 2022.

### 2.10. Cell Culture and In Vitro Cytotoxicity Using MTT Assay

#### 2.10.1. Cell Culture

The human leukemia monocyte cell line (THP-1) was purchased from ATCC^®^, USA, and consistently cultured in Roswell Park Memorial Institute medium (RPMI-1640 Medium) supplemented with 10% (*v*/*v*) fetal bovine serum (FBS), 2 mM L-glutamine, 100 units mL^−1^ penicillin G. sodium, 100 units mL^−1^ streptomycin sulphate, and 250 ng mL^−1^ amphotericin B. All reagents were supplied by Lonza (Basel, Switzerland). THP-1 cells were treated with 100 ng mL^−1^ phorbol-12-myristate-13-acetate (PMA) for 48 h. Subsequently, the differentiated macrophages were cultured in fresh RPMI-1640 medium for 24 h before experimental application [49]. All cells were maintained at subconfluency at 37 °C in a humidified atmosphere with 5% CO_2_. For subculturing, monolayer cells were harvested after trypsin/EDTA treatment at 37 °C and were used when confluence reached 75%.

#### 2.10.2. MTT Assay

Next, the MTT (3-[4,5-dimethylthiazol-2-yl]-2,5 diphenyl tetrazolium bromide) method was used to assay the in vitro cytotoxicity of MPI nanoparticles. MTT was obtained from Merck KGaA (Darmstadt, Germany). Cells (1 × 10^4^ cells/well) were seeded in a serum-free medium in a flat-bottom 96-well microplate. They were then exposed to a 20 µL nanoprotein sample per well at different concentrations of 1.5, 3, 6.25, 12.5, 25, 50, 100, 150, 200, and 250 μg mL^−1^ for 48 h at 37 °C in a humidified atmosphere with 5% CO_2_. After incubation, the media were discarded, and 40 µL of MTT solution was added to each well, followed by a further 4 h incubation. The MTT crystals were dissolved in 180 µL of acidified isopropanol/well. The plates were shaken at room temperature, and the absorbance was measured at 570 nm using a microplate ELISA reader (FLUOstar OPTIMA, BMG LABTECH GmbH, Ortenberg, Germany). The IC_50_ was obtained using non-linear regression analysis of the concentration-response curve and OriginPro2024 version (Learning Edition). The absorbance of wells filled with media alone was used as a blank, and untreated control wells were seeded with cells incubated without MPI nanoparticles. Cell viability percentage (Cv %) was calculated using Equation (1):

(1) $$\mathrm{Cv}\;\left(\%\right)=\frac{At}{Ac}\times 10$$

where (*Ac*) is the absorbance of the control (untreated cells), and (*At*) is the absorbance of the tested sample, as previously described [50]. The cell cytotoxicity % was calculated using Equation (2):

(2) % Cell cytotoxicity =100− % Cell viability

Each concentration was repeated three times, and the average was calculated. All phases of this experiment were carried out at the NRC and the Creative Egyptian Biotechnologists (C.E.B) Lab.

#### 2.10.3. Cytokine Assay by ELISA

The IL-6 and TNF-α levels in the matrix were measured using commercial ELISA kits. Briefly, THP-1 cells were pre-incubated in a 12-well plate at a density of 1.0 × 10^6^ cells per well and maintained for 24 h in a 5% CO_2_ incubator at 37 °C. Culture media were collected and transferred into 96-well plates coated with 100 μL of antibody solutions of IL-6 and TNF-α at 37 °C for 60 min. Next, 100 μL of horseradish peroxidase (HRP) solution was added after four washes, and the plates were incubated at 37 °C for 40 min. Next, 100 μL of 3,3′,5,5′ tetramethylbenzidine (TMB) substrate solution was added after four washes, and the plates were incubated at 37 °C in the dark for 15 min. Finally, the absorbance at 450 nm was read on a microplate ELISA reader (FLUOstar OPTIMA, BMG LABTECH GmbH, Ortenberg, Germany) within 5 min after the addition of a stop solution. The control group was incubated without the PBNPs, and the treated samples were incubated at a low PBNP concentration of 50 µg mL^−1^. Cytokine levels were calculated according to standard curves [50]. All tests were conducted in triplicate.

## 3. Results and Discussion

### 3.1. Proximate Composition

In Table 1, we present the proximate compositions of Moringa seed flour and Moringa seed cake flour. The data revealed that Moringa seed cake flour contains higher moisture, ash, fiber, protein, and carbohydrate levels. Furthermore, the chemical composition of moringa seed flour aligns with the previously reported values. These elevated values can be attributed to the displacement of oil from Moringa seed flour [51]. Moringa seed cake flour’s moisture content, ash, carbohydrates, and fiber content closely aligned with the findings reported by Saa et al. (2022). Moringa seed cake flour contains a high protein content, which is consistent with the literature values [52]. Comparatively, our Moringa seed cake flour had a protein content of 54.20%, significantly higher than the 45.8% reported by Cattan et al. (2022). However, the oil content was lower at 3.17%, compared to their reported 11.20%. This discrepancy may be due to the use of different oil extraction methods [53]. The isolated lyophilized protein exhibited a purity of 92.31%, which is aligned with the >90% purity observed by Jain et al. (2019) [11]. Variations in seed composition may be attributed to differences in variety, climate, maturity stage, harvest time, and the extraction method used for the seed.

### 3.2. Characterization of MPI and PBNPs Nanoparticles

#### 3.2.1. The Morphology and Particle Size

**Figure 1:**
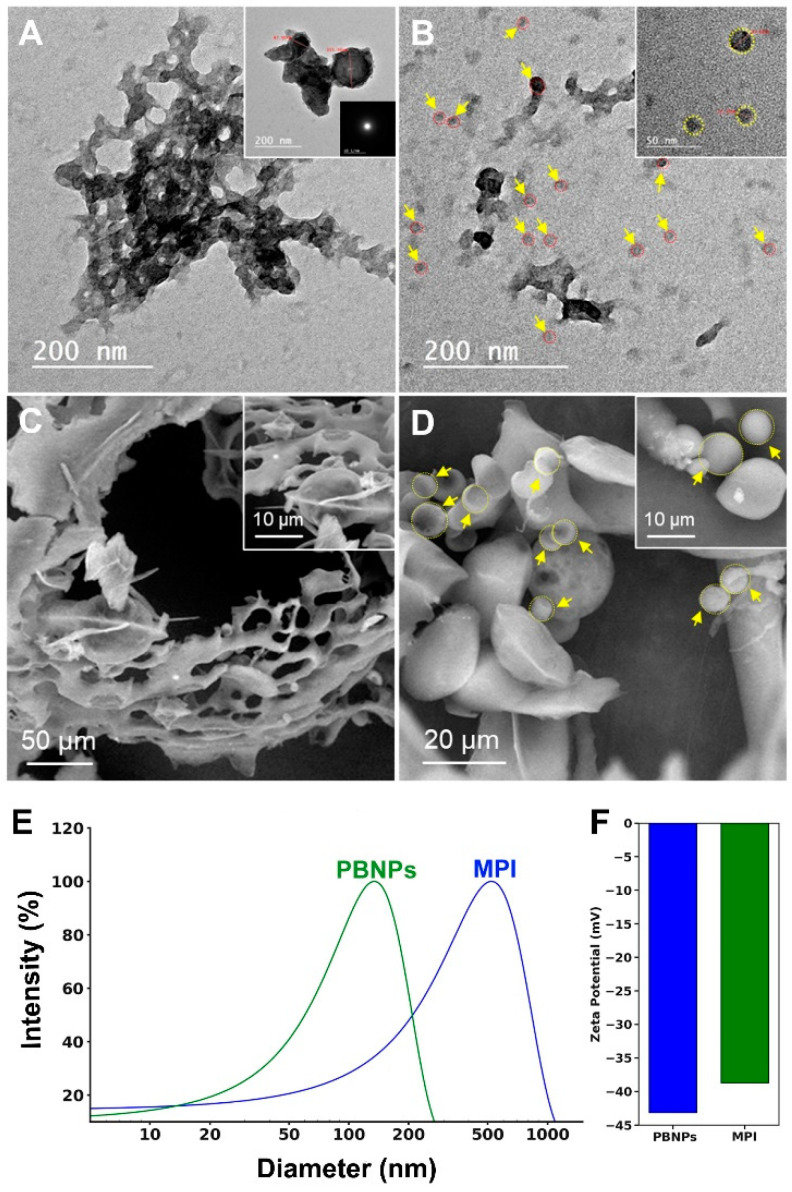
Characterization of the morphology, particle size, and zeta potential of MPI and PBNPs: (**A**) HR-TEM image of inert MPI; (**B**) HR-TEM image of inert PBNPs; (**C**) HR-SEM image of inert MPI; (**D**) HR-SEM image of inert PBNPs; (**E**) DLS analysis of MPI (before ultrasonication) and PBNPs (after ultrasonication); (**F**) zeta potential measurements of PBNPs (blue) and MPI (green).

The morphological characteristics of Moringa protein isolate (MPI) and protein-based nanoparticles (PBNPs) were examined using TEM and SEM imaging, revealing significant size and shape variations. The TEM images in Figure 1A,B display Moringa protein isolate (MPI) and protein-based nanoparticles (PBNPs), with particle sizes ranging from 155.36 to 12.35 nm, respectively, which were approximately 60% smaller than the values obtained by DLS. This difference can be attributed to the shrinkage caused by the cast-drying process and vacuum environment used in TEM imaging [54]. The SEM images of MPI and PBNPs presented in Figure 1C,D clearly illustrate a non-specific branched shape. In contrast, the SEM results for PBNPs showed a sticky or almost spherical shape.

The average particle size and zeta potential (ζ) are important indicators that reflect the stability of the nanoparticles in dispersion. The MPI DLS results showed an average particle size of 521.4 ± 0.51 nm, indicating the presence of large aggregates [52]. After ultrasonication, the average particle size was 134.3 ± 0.47 nm of the PBNPs, as presented in Figure 1E. The particle size decreased significantly after ultrasonication, which could be attributed to the disruption of noncovalent interactions under high-intensity ultrasonication and the conversion of large aggregates into smaller nanoparticles [55,56]. The initial zeta (ζ) potential (mV) result of the MPI dispersion was approximately −38.70 mV, and the PBNPs exhibited a favorable combination of stick-like and spherical shapes, with a zeta potential of −43.15 mV, as shown in Figure 1F. This high absolute zeta potential value (greater than ±30 mV) suggests excellent particle stability, indicating strong electrostatic repulsion between the nanoparticles, which prevents aggregation. We observed a decrease in the zeta potential to −43.15 mV following ultrasonication. This reduction is attributed to the acoustic treatment, which helps to bury the non-polar groups and thereby increases the polarity and solubility due to the appearance of ionized groups on the surface. The negative zeta potentials confirmed the strong electrostatic repulsion between the nanoparticles, further supporting their stability [57].

In addition, the polydispersity index (PDI) decreased significantly from 0.258 in the initial sample to 0.220 after ultrasonication. This reduction in PDI, coupled with the decrease in mean particle diameter from 521.4 nm to 134.3 nm, indicates that the ultrasonication technique successfully reduces the size and homogenizes the MPI, leading to a more consistent distribution of PBNPs, as shown in Table S1.

#### 3.2.2. X-ray Diffraction (XRD) and EDX Analysis

**Figure 2:**
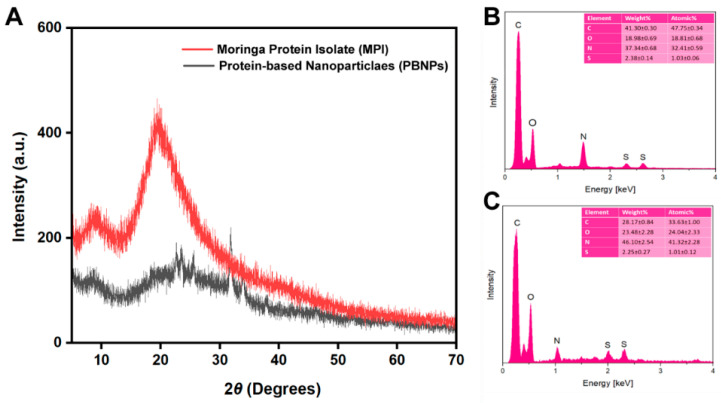
X-ray diffraction (XRD) and EDX analyses: (**A**) XRD analysis of *M. oleifera* seeds. In red, the moringa protein isolate (MPI), and in black, the protein-based nanoparticles (PBNPs); (**B**) EDX elemental composition analysis of MPI; (**C**) EDX elemental composition analysis of PBNPs.

X-ray scattering techniques are commonly used to obtain information on the structural properties of biopolymers, including proteins [58]. The structural characteristics of MPI and PBNPs were studied using XRD (Figure 2A). MPI presented two distinct diffraction peaks at 2θ. It showed a high peak at approximately 19.50° and a small peak at approximately 8.8°, which also refer to the secondary conformation of the protein α-helix and β-sheet, respectively [59,60]. These results are very close to the XRD results for soybean proteins obtained in previous studies [47,61]. In the analysis of nanoparticle composition, the observed crystalline structures were identified as integral components of the nanoparticles. At the same time, we found that the peaks became weak in the XRD pattern of the PBNPs by forming new peaks at 2θ values of approximately 24° and a sharp peak at 32°. This indicates an increase in the overall degree of crosslinking in the case of PBNPs due to the formation of more secondary bonds, as shown in the FT-IR results, ensuring the rearrangement of the protein structure by converting MPI into a nanostructure [62,63].

Moreover, EDX was used to detect the elemental location and surface composition of MPI and PBNPs after ultrasonication. The analysis of the EDX spectrum indicated that the main components were the same in both proteins and nanoproteins, suggesting that the protein was transformed into a nanoform through self-assembly. Moreover, the EDX analysis revealed the presence of carbon, nitrogen, oxygen, and sulfur. There was a noticeable change in the proportion of carbon and nitrogen, a moderate change in the proportion of oxygen, and a very slight change in the proportion of sulfur in MPI and PBNPs. The changes in the elemental composition during quantitative microelectrochemical analysis (EDX) are shown in Figure 2B,C, which may be attributed to the ultrasonication process [64].

#### 3.2.3. Fourier-Transform Infrared Spectroscopy (FT-IR)

**Figure 3:**
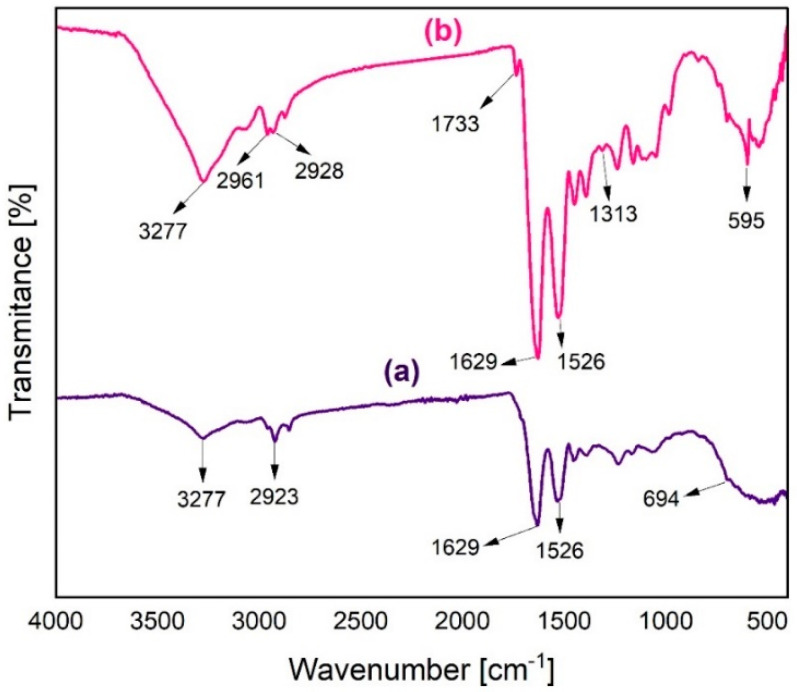
FT-IR spectrum of PI and PBNPs produced from *M. oleifera* seeds: (**a**) Moringa protein isolate (MPI); (**b**) protein-based nanoparticles (PBNPs).

FT-IR analysis was used to examine the primary functional groups present in MPI and PBNPs Figure 3. The FT-IR spectrum of MPI has three characteristics: absorption peaks located at 3277, 1629, and 1526 cm^−1^, which are attributed to the O-H stretching vibration, while the other two peaks are located in the amide II band, which is due to the N-H bending vibration (40–60%) and stretching vibration. C-N (18–40%), which is evidence of peptide bonds forming the primary backbone of proteins [65,66,67]. These three peaks were also observed for the PBNPs, with slight peak shifts. This means that some changes in the amino acid contributions may occur due to different locations and densities of the functional groups when transferred to the nanostructure [68].

New adsorption peaks were detected at 1733, 1313, and 595 cm^−1^, which were assigned to the characteristic adsorption band of aldehyde (C=O stretching aldehyde) and the symmetric stretching of COO^−^ in carboxylic amino acids [69]. The sharp peaks occurring at 595 cm^−1^ are the result of out-of-plane bending of the N-H bond, originally in the V band of the amide group in Moringa seed nanoprotein [13,70].

The increase in the peak intensity in the case of PBNPs serves as evidence for the transformation of MPI into a nanostructured form. This nanostructure is characterized by the dense presence of active groups [71]. Furthermore, an adsorption peak was observed at 3277 cm^−1^, indicating the formation of additional hydrogen bonds. This provides further proof that the protein undergoes a transition into a nanostructure due to self-assembly and potentially as a result of cross-linking caused by hydrophobic amino acids. FT-IR analysis showed that PBNPs were mainly formed through electrostatic, hydrogen, and hydrophobic interactions [72,73].

### 3.3. Influence of pH on MPI Charge, Isolation Efficiency, and Stability of PBNPs

**Figure 4:**
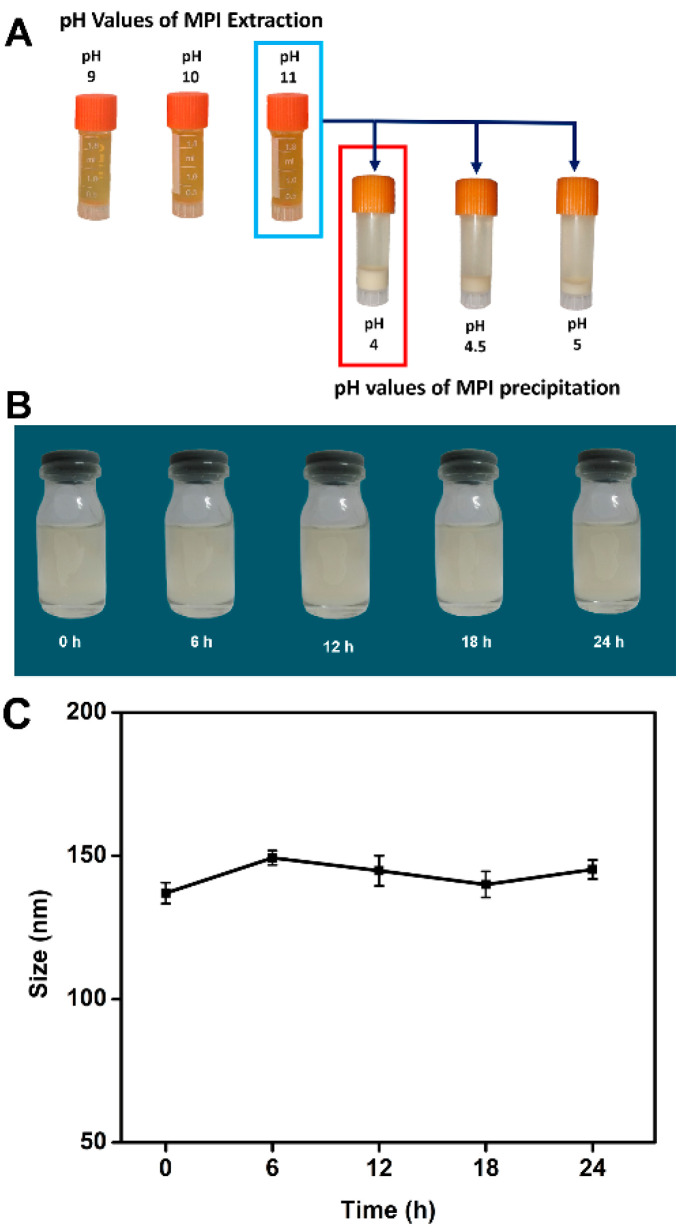
Influence of pH on the MPI charge, isolation efficiency, and stability of PBNPs. (**A**) Influence of pH on MPI charge and isolation efficiency; (**B**) stability of PBNPs over 24 h; (**C**) the DLS analysis of the PBNPs over different time intervals.

Due to the ionization of the protein surface groups, proteins are usually positively charged in an acidic solution and negatively charged in an alkaline solution. When the zeta potential is 0 mV at a certain pH value, the electrostatic repulsion between protein molecules is reduced, and consequently, the protein precipitates [74]. The first step in the protein isolation process is the extraction step, which is highly pH-dependent. Higher pH values are associated with higher yields. However, the raw material is susceptible to oxidation under strongly alkaline conditions, such as pH 12, with a dark brown color, unlike the natural yellow color characteristic of the protein extract solutions. A high pH may also lead to the destruction of proteins and the formation of lysine–alanine complexes, leading to a decrease in biological value and possibly the formation of toxic compounds [75]. Therefore, extraction at pH 11, 10, and 9 was chosen as a compromise to obtain as much protein as possible while retaining its bioactive properties. The naked-eye observations of the three tubes shown in Figure 4A indicate a slight difference in the intensity of the yellow color, with the tube at pH 11 appearing more intense in color, which can be attributed to greater solubilization of the protein. For the isoelectric protein precipitation, the pH of the protein extract obtained at pH 11 was adjusted to 4, 4.5, and 5. The total wet mass (the precipitated portion at the end of the process) produced when the pH was lowered was used as an initial visual indicator of the amount of precipitated protein. The largest wet mass fraction was found at pH 4 and the lowest at pH 5; these results are consistent with those reported in the study conducted by González Garza and Nancy Gisela, et al. (2017) [38].

The stability of PBNPs in solution is important for biological applications [25,76]. MPI was used to prepare the PBNPs. Several images of the PBNP solution were captured every 2 h, as presented in Figure S1, and every 6 h, as illustrated in Figure 4B, to monitor the stability of the nanoparticles in the solution over a 24 h period. No precipitation of the PBNPs was observed. Several images of the dissolved PBNPs were also captured once daily for 10 days. As can be seen in Figure S2, no significant differences were observed. Dynamic light scattering (DLS) analysis of the PBNPs was conducted to confirm the stability and solubility of the nanoproteins, as shown in Figure 4C. This was attributed to the good stability and solubility of the Moringa nanoprotein. Ultrasonication can improve the functional characteristics related to the increased solubility of PBNPs.

## 4. Molecular Weight of MPI and PBNPs

Under non-reducing conditions, the proteomic profile of MPI showed high-intensity protein bands of 10 kDa, less intense protein bands of up to 25 kDa, and much less intense protein bands of up to approximately 55 kDa. Meanwhile, the PBNPs before and after sonication exhibited molecular weights of 10 kDa or less, as shown in Figure S3. The protein profile observed in our study was almost identical to that previously reported for Moringa seed proteins [39,77]. This result is consistent with those previously reported [78,79]. Most Moringa proteins have relatively low molecular weights, ranging between 3.4–20 kDa. The *M. oleifera* seed protein candidate used in this study has potential applications in the preparation of nanoparticles.

### 4.1. Cytotoxicity Assay

**Figure 5:**
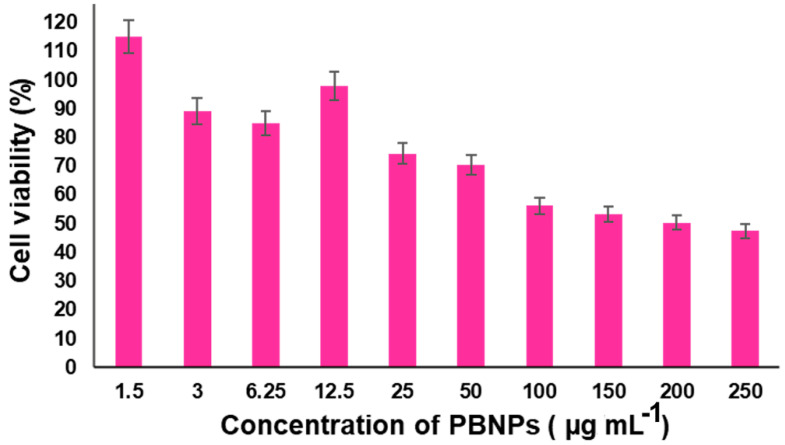
Dose-response MTT assay. Effects of different concentrations of PBNP on THP-1 cell viability (%).

The cytotoxic effect of nanoparticles was evaluated by MTT, a colorimetric assay that measures cell metabolic activity based on the reduction of tetrazolium salt into insoluble formazan crystals by the mitochondrial reductase enzyme of viable cells, as illustrated in Figure S4. The amount of formazan crystals produced determines the cell viability [80,81]. The effects of different concentrations of PBNPs, i.e., 1.5, 3, 6.25, 12.5, 25, 50, 100, 150, 200, and 250 μg mL^−1^, on the cell viability of the THP-1 cells were studied. The results showed that low concentrations of PBNPs (1.5, 3, 6.25, and 12.5 μg mL^−1^) were non-toxic to THP-1 cells and contributed to increased cell viability. As the concentration of PBNPs increased, a slight decrease in cell viability was observed in Figure 5. These results indicate that PBNPs produced from *M. oleifera* seeds are non-toxic to THP-1 cells at low concentrations. However, compared with PBNPs produced from Moringa seeds, those produced from soybean seeds showed more cytotoxic effects [82]. These results indicate the bright future of *M. oleifera* seed protein nanoparticles in the medical field [76,83,84]. The cell viability % of THP-1 cells versus the log concentration and IC50 value of PBNPs was obtained using a non-linear regression analysis of the concentration–response curve from Moringa protein nanoparticles, as demonstrated in Figure S4.

### 4.2. Cytokine Expression Levels

Pro-inflammatory cytokines such as TNF-α and IL-6 are linked to cancer [85]. TNF-α is the main cytokine involved in inflammatory reactions and the first cytokine used in cancer treatment that promotes antitumor activity through inflammatory and immune responses and induces cancer cell death [86]. IL-6 stimulates systemic immune responses and creates an immune suppression environment that protects cancer cells and plays a role in various cancers, including solid tumors and hematomas [87]. We investigated the effects of *M. oleifera* seed protein nanoparticles on cytokine secretion (TNF-α and IL-6) in THP-1 cells using ELISA kits. To ensure that the observed effects were not skewed by cytotoxicity, we conducted the cytokine assay at a low PBNP concentration of 50 µg mL^−1^. The results showed an increase in TNF-α production of 89.23 ± 0.76 pg mL^−1^ compared with the control, which was 78 ± 1.78 pg mL^−1^. Conversely, the IL-6 levels decreased to 88 ± 0.92 pg mL^−1^ compared with the control, which was 113 ± 3.40 pg mL^−1^, as shown in Table 2. These findings indicate that increased levels of TNF-α could have prominent pro-inflammatory effects and can inhibit cancer cell proliferation via immunomodulation [88,89]. The reduction in IL-6 levels suggests a potential decrease in the immunosuppressive environment, which could further support the antitumor activity of TNF-α by promoting a more effective immune response against cancer cells. Our data provide new insights into the immunomodulatory effects of *Moringa oleifera* seed protein nanoparticles and highlight their potential as a therapeutic agent in cancer treatment. Further studies are needed to elucidate the precise mechanisms by which PBNPs modulate cytokine levels and their overall impact on cancer progression and immune response.

## 5. Conclusions

In this study, we successfully produced protein nanoparticles from *Moringa oleifera* seeds using an innovative ultrasonication solubilization approach. The resulting protein nanoparticles, with an average size of 134.3 ± 0.47 nm, were characterized using advanced techniques such as DLS, TEM, and SEM. This production method, achieved without enzymatic hydrolysis, positions *M. oleifera* as an excellent source of plant-derived protein nanoparticles. Our in vitro experiments demonstrated a concentration-dependent reduction in THP-1 cell viability, with significant cytotoxic effects observed at a concentration of 206.5 μg mL^−1^. Importantly, at lower concentrations, PBNPs from *M. oleifera* seed proteins modulated cytokine secretion, evidenced by an increase in TNF-α levels to 89.23 ± 0.76 pg mL^−1^ and a decrease in IL-6 to 88 ± 0.92 pg mL^−1^. These findings accentuate the potential of *M. oleifera* seeds in producing protein nanoparticles that can be leveraged in biomedical research. The modulation of cytokine levels by these nanoparticles suggests a mechanism of action that includes significant immunomodulatory effects, which could contribute to antitumor activity. However, further studies are required to confirm these results. Interestingly, this study highlights the therapeutic potential of *M. oleifera* protein nanoparticles, paving the way for further research into their applications in cancer treatment and other areas of biomedicine. By providing a novel method for producing plant-based protein nanoparticles and demonstrating their biological activity, our research contributes valuable insights into the development of new, natural therapeutic agents.

## Figures and Tables

**Table 1:** Proximate composition of Moringa seed flour and Moringa seed cake flour (% *w*/*w*).

Parameter	Moringa Seed Flour	Moringa Seed Cake Flour
Moisture	5.35 ± 0.15	6.93 ± 0.08
Ash	3.55 ± 0.05	4.72 ± 0.08
Protein	32.00 ± 0.20	54.20 ± 0.10
Lipids	36.20 ± 0.30	3.17 ± 0.17
Fibers	7.00 ± 0.20	9.12 ± 0.08
Carbohydrates	15.90 ± 0.10	20.87 ± 0.83

Data indicates ± standard deviation (n = 3).

**Table 2:** The effect on the secretion of cytokines (TNF-α and IL-6) from THP-1 cells after treatment with PBNPs at low concentration.

Test	IL-6 (pg mL^−1^)	TNF-α (pg mL^−1^)
Control	113 ± 3.40	78 ± 1.78
Treated	88 ± 0.92	89.23 ± 0.76

Data indicates ± standard deviation (n = 3).

## Data Availability

The datasets generated and/or analyzed during the current study are available from the corresponding author upon reasonable request.

## Supplementary Materials

The following supporting information can be downloaded at: https://www.mdpi.com/article/10.3390/nano14151254/s1, Figure S1: PBNP dispersion stability every two hours for 24 h following ultrasonication; Figure S2: PBNP dispersion stability for 10 days following ultrasonication; Figure S3: Molecular weight of MPI and PBNPs and SDS page: Marker (M), Crude Protein, PBNPs Before Sonication, and PBNPs after sonication; Figure S4: Dose-response MTT assay: The viability assay, the horizontal dashed line indicates 50% viability, and the vertical dashed line marks the IC50 value of 2.315 (conc. 206.5 μg mL^−1^); Table S1: Characterization of moringa protein isolate and protein-based nanoparticles. Details of the proximate composition procedure in the Materials and Methods section are provided in the Supporting Information (SI) section.

## Abbreviations

% *w*/*w*, % weight/weight; AOAC, Association of Analytical Communities; DLS, dynamic light scattering; DMSF, defatted Moringa seed flour; EDX, energy-dispersive X-ray analysis; ELISA, enzyme-linked immunosorbent assay; FT-IR, Fourier-transform infrared spectroscopy; HR-TEM, high-resolution TEM; (kDa), kilodaltons; *M. oleifera, Moringa oleifera*; MPI, Moringa protein isolate; MTT, (3-[4,5-dimethylthiazole-2-yl]-2,5-diphenyltetrazolium bromide); MWCO, molecular weight cut-off; PBNPs, protein-based nanoparticles; RPMI-1640, Roswell Park Memorial Institute media; PMA, phorbol-12-myristate-13-acetate SEM, scanning electron microscopy; SDS-PAGE, sodium dodecyl sulfate–polyacrylamide gel electrophoresis; TEM, transmission electron microscopy; THP-1, the human monocyte cell line; XRD, X-ray diffraction.
