# Supplementary material for: Ultrasonication-Assisted Green Synthesis and Physicochemical and Cytotoxic Activity Characterization of Protein-Based Nanoparticles from Moringa oleifera Seeds
Source: Nanomaterials (Basel). 2024 Jul 26;14(15):1254. doi: 10.3390/nano14151254 (PMC11313732; doi:10.3390/nano14151254)
Supplement: Supplementary file 1 [file nanomaterials-14-01254-s001.zip › nanomaterials-3055390-supplementary.pdf]

Supplementary Material

Article

Ultrasonication-Assisted Green Synthesis and Physicochemical and Cytotoxic Activity Characterization of Protein-Based Nanoparticles from Moringa Oleifera Seeds

Amany Abd El-Shafy Abd El-Kader Nafeh [1], Ibrahim Mohamed Abd El-Aleem Mohamed [1], and Mohamed Frahat Foda [1,2,*]

1 Department of Biochemistry, Faculty of Agriculture, Benha University, Moshtohor, Toukh 13736, Egypt;
2 National Key Laboratory of Crop Genetic Improvement, College of Life Science and Technology, Huazhong Agricultural University, Wuhan 430070, China
* Correspondence: m.frahat@mail.hzau.edu.cn

2. Materials and Methods

2.5. Proximate composition

2.5.1. Moisture, ash, and crude fiber content

Initially, moisture content was assessed utilizing the oven-drying method. Briefly, a 20 g sample was placed in several dishes previously weighed in an oven and dried at 105°C for 12 h. Then, the samples were cooled in a desiccator, which resulted in a final dry weight of about 18.93 g of moringa seed flour and 18.62 g of moringa seed cake flour, and the difference in weight was used to calculate the moisture content in the samples. Subsequently, ash content was assessed by analyzing the inorganic residues following ignition or complete oxidation of organic matter. In this process, 2 g of previously dried samples underwent a 3 h muffle treatment at 550°C. The ash % was determined by weighing the resulting inorganic residue. Finally, crude fiber content was determined by calculating the percentage of crude fiber after ignition as the dry residue loss. This process involved digesting 2 g of previously dried samples with sulfuric acid and sodium hydroxide under specific conditions. The weight difference after calcination indicated the fiber content ($n=3$).

2.5.2. Fat content

The flour was obtained based on continuous solvent extraction using a Soxhlet apparatus for 6 h. The lipid percentage was calculated from the weight of the container with the extracted lipids using hexane (95%) minus the weight of the container without fat in constant weight with three replicates ($n=3$).

2.5.3. Protein content

The protein content in the flour was determined based on the total nitrogen by the Kjeldahl method. Briefly, 0.5 g of samples were digested with sulfuric acid in the presence of catalysts; the digested samples were neutralized with concentrated NaOH to collect the resulting ammonium, and subsequently, the nitrogen present in the sample was determined through titration. To calculate the % protein in the sample, nitrogen-to-protein conversion was 6.25 factor with three replicates ($n=3$).

2.5.4. Carbohydrate contents

The carbohydrate content was calculated using the following Eq. **(1)** [15]:

$$\%\text{Carbohydrate} = 100 - (\%\text{Moisture} + \%\text{Ash} + \%\text{Fiber} + \%\text{Protein} + \%Fat). \quad \textbf{(1)}$$

Table S1 Characterization of moringa protein isolate and protein-based nanoparticles.

Samples	Mean Diameter (nm)	Zeta (ζ) potential (mV)	Polydispersity Index (PDI)
**Moringa Protein isolate (MPI)**	521.4	-38.70	0.258
**Protein-based nanoparticles (PBNPs)**	134.3	-43.15	0.220

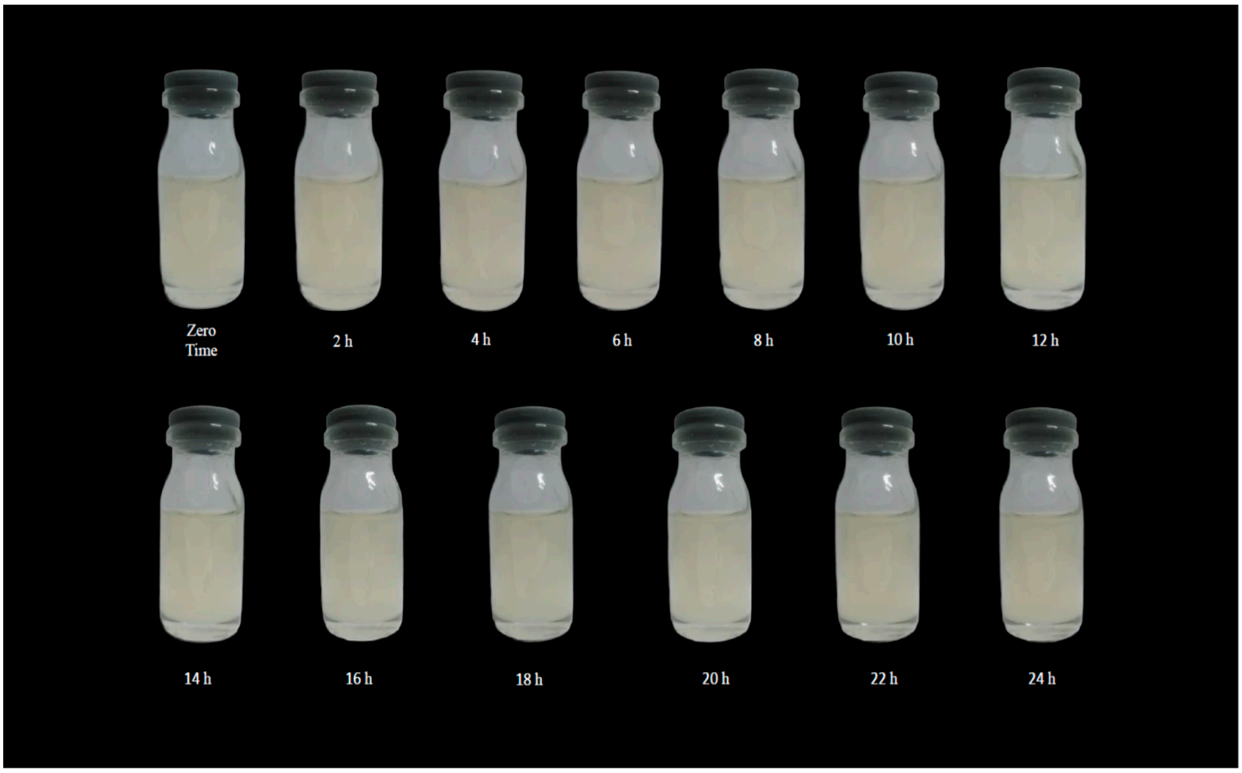

Fig. S1 PBNP dispersion stability every two hours for 24 hours following ultrasonication.

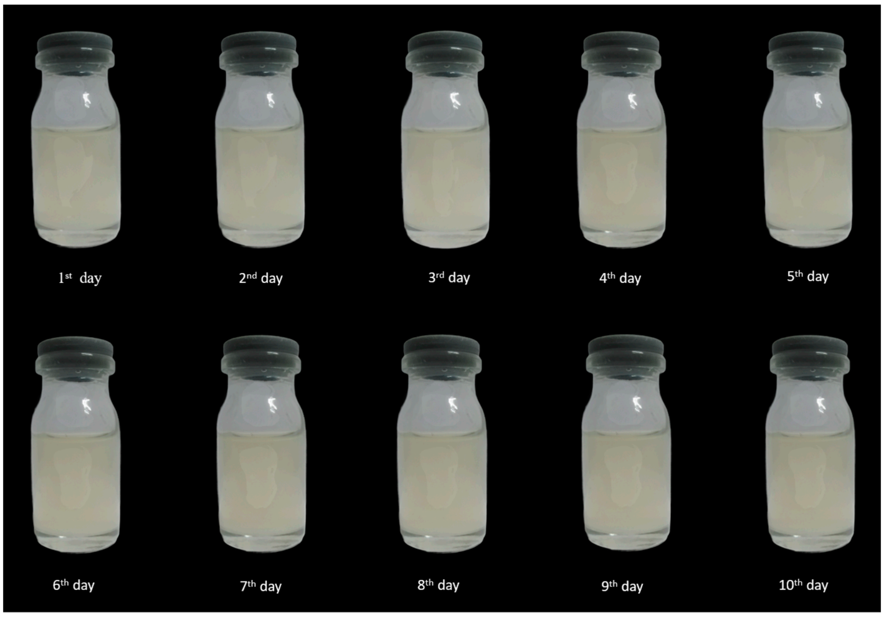

Fig. S2 PBNP dispersion stability for 10 days following ultrasonication.

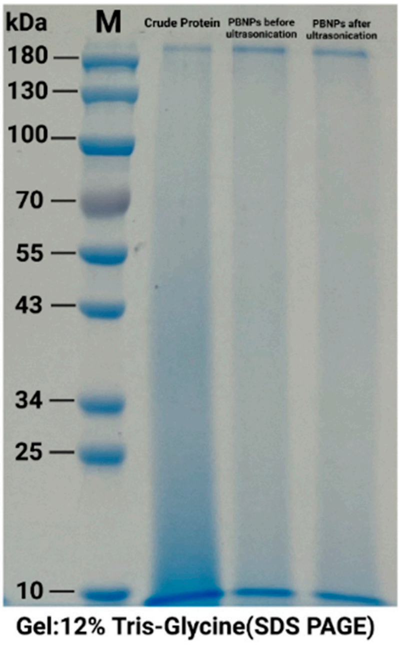

Fig. S3 Molecular weight of MPI and PBNPs and SDS page: Marker (M), Crude Protein, PBNPs Before Sonication, and PBNPs after sonication.

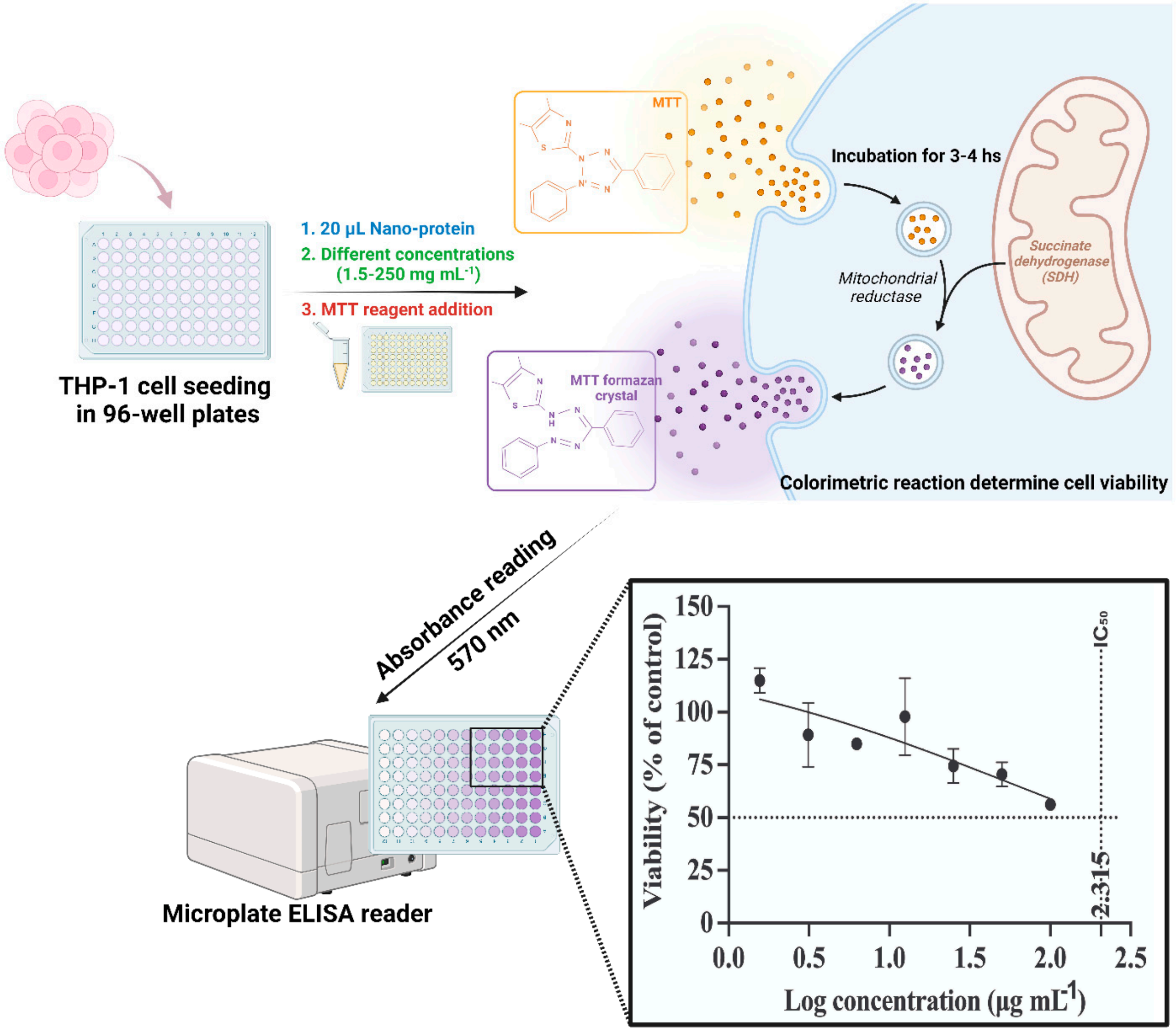

Fig. S4 Dose-response MTT assay: The viability assay, the horizontal dashed line indicates 50% viability and the vertical dashed line marks the IC_{50} value of 2.315 (conc. 206.5 µg mL-1).
